# DNA methylation and hormone receptor status in breast cancer

**DOI:** 10.1186/s13148-016-0184-7

**Published:** 2016-02-16

**Authors:** Elizaveta V. Benevolenskaya, Abul B. M. M. K. Islam, Habibul Ahsan, Muhammad G. Kibriya, Farzana Jasmine, Ben Wolff, Umaima Al-Alem, Elizabeth Wiley, Andre Kajdacsy-Balla, Virgilia Macias, Garth H. Rauscher

**Affiliations:** Department of Biochemistry and Molecular Genetics, College of Medicine, University of Illinois at Chicago (UIC), M/C 669, 900 S. Ashland Ave., Chicago, 60607 IL USA; Department of Genetic Engineering and Biotechnology, University of Dhaka, Dhaka, Bangladesh; Department of Health Sciences, The University of Chicago, Chicago, USA; Loyola University, Chicago, USA; Division of Epidemiology and Biostatistics, School of Public Health, University of Illinois at Chicago (UIC), M/C 923, Chicago, 60612 IL USA; Department of Pathology, University of Illinois at Chicago, Chicago, USA

**Keywords:** DNA methylation, Breast cancer, ER/PR hormone receptor status

## Abstract

**Background:**

We examined whether differences in tumor DNA methylation were associated with more aggressive hormone receptor-negative breast cancer in an ethnically diverse group of patients in the Breast Cancer Care in Chicago (BCCC) study and using data from The Cancer Genome Atlas (TCGA).

**Results:**

DNA was extracted from formalin-fixed, paraffin-embedded samples on 75 patients (21 White, 31 African-American, and 23 Hispanic) (training dataset) enrolled in the BCCC. Hormone receptor status was defined as negative if tumors were negative for both estrogen and progesterone (ER/PR) receptors (*N* = 22/75). DNA methylation was analyzed at 1505 CpG sites within 807 gene promoters using the Illumina GoldenGate assay. Differential DNA methylation as a predictor of hormone receptor status was tested while controlling for false discovery rate and assigned to the gene closest to the respective CpG site. Next, those genes that predicted ER/PR status were validated using TCGA data with respect to DNA methylation (validation dataset), and correlations between CpG methylation and gene expression were examined. In the training dataset, 5.7 % of promoter mean methylation values (46/807) were associated with receptor status at *P* < 0.05; for 88 % of these (38/46), hypermethylation was associated with receptor-positive disease. Hypermethylation for *FZD9*, *MME*, *BCAP31*, *HDAC9*, *PAX6*, *SCGB3A1*, *PDGFRA*, *IGFBP3*, and *PTGS2* genes most strongly predicted receptor-positive disease. Twenty-one of 24 predictor genes from the training dataset were confirmed in the validation dataset. The level of DNA methylation at 19 out 22 genes, for which gene expression data were available, was associated with gene activity.

**Conclusions:**

Higher levels of promoter methylation strongly correlate with hormone receptor positive status of breast tumors. For most of the genes identified in our training dataset as ER/PR receptor status predictors, DNA methylation correlated with stable gene expression level. The predictors performed well when evaluated on independent set of samples, with different racioethnic distribution, thus providing evidence that this set of DNA methylation biomarkers will likely generalize to prospective patient samples.

**Electronic supplementary material:**

The online version of this article (doi:10.1186/s13148-016-0184-7) contains supplementary material, which is available to authorized users.

## Background

Breast cancer has traditionally been described by histopathological staging based on size, degree of invasiveness, and lymph node metastasis and by immunochemical analysis of the epidermal growth factor receptor HER2 and the estrogen (ER) and progesterone (PR) receptors. Recently, there has been an increased awareness of the potential influence of socioeconomic and psychosocial factors on breast cancer aggressiveness characteristics [1–5]. One mechanism by which these processes might exert their effects on activity of breast cancer genes is through epigenetic alterations, including DNA methylation. Therefore, addition of classification based on DNA methylation and gene expression might improve prognostic prediction to therapeutic response or survival.

Previous studies using established cancer cell models showed that tumor evolution includes genome-wide loss of DNA methylation (hypomethylation) as well as increase in promoter methylation at CpG islands (promoter hypermethylation) [6]. Genes involved in specific biological pathways have been recognized to be methylated at their promoters in various types of cancer, including breast cancer [7]. Distinctive patterns of promoter methylation have been reported previously for ER/PR-positive versus ER/PR-negative tumors [8–10]. ER/PR-negative tumors are of particular interest because they tend to be the most aggressive form and lack targets for hormone therapy. Therefore, these new DNA methylation-based characteristics had a potential to contribute prognostic value in breast cancer management. Prior studies using panels of DNA methylation markers, however, are plagued by lack of reproducibility, in part because these studies tend to focus on the top-most performing markers [11], as opposed to genome-wide association. The reproducibility was likely varied from study to study due to random error associated with commonly used small sample size. The prevalence of certain markers in particular cohort populations was not taken into account, as race and ethnicity were either not reported or lacking Hispanic and African-American patient population [8–10, 12].

The aims of our analyses were (1) to identify a set of gene DNA methylation markers predictive of ER/PR status in a training dataset of invasive breast cancer samples from an ethnically diverse patient cohort (the Breast Cancer Care in Chicago (BCCC) study); (2) to validate DNA methylation markers identified in the training data using a different, publically available validation dataset; and (3) to associate these DNA methylation markers with corresponding gene expression changes. Through this approach, our goal was to identify and validate a set of gene methylation markers that may play an etiologic role in breast cancer subtypes.

## Results

### The level of DNA methylation is higher in ER/PR-positive tumors in the training dataset

We tested if associations can be drawn between the levels of DNA methylation and hormone receptor ER/PR status in an ethnically diverse patient cohort with invasive breast cancer disease. The cohort included the ER/PR-positive group with tumors that were either ER or PR positive and the ER/PR-negative group with tumors that were both ER and PR negative. Patients with ER/PR-positive tumors were similar to patients with ER/PR tumors with regard to age at diagnosis, race/ethnicity, stage at diagnosis, and family history of breast cancer (Table 1).

**Table 1:** Patient and tumor characteristics by hormone receptor status in the BCCC dataset

	Total (*N* = 75)		ER/PR positive	
	*N*	%	%	*P* value (chi-square test)
Age at diagnosis				0.88
<50	20	27	75	
50–59	20	27	70	
60–79	35	47	69	
Race/ethnicity				0.44
nH Black	31	41	65	
nH White	21	28	81	
Hispanic	23	31	70	
Pathological stage				0.39
1	23	31	83	
2	33	44	67	
3	18	24	61	
4	1	1	100	
Histologic grade				0.10
Low	5	7	100	
Moderate	31	42	77	
High	38	51	61	
1st degree familial breast cancer				0.25
None	50	68	68	
Moderate	18	24	67	
Strong (<50/multiple affected)	6	8	100	

**Fig. 1:**
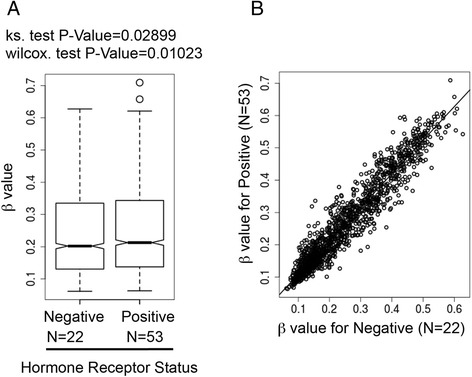
Distribution of DNA methylation between ER/PR-negative and ER/PR-positive samples in the training dataset (BCCC). **a** Box plot of mean *β* values. The level of methylation at each CpG site was defined by *β* values. *β* values close to 0 indicated low level of DNA methylation, and *β* values close to 1 indicated high level of DNA methylation. The next levels of analysis were conducted at the gene level. Mean *β* values were averaged for all CpG sites on the array for each individual gene. Statistical significance of difference in *β* values for each gene between the two groups was determined by ks and Wilcox tests. **b** Scatter plot analysis of mean β values

The group of ER/PR-positive tumors had significantly higher mean *β* values than the group of hormone-negative tumors (Fig. 1a). The scatter plot analysis showed that *β* values for many genes were shifted relative to the identity line (Fig. 1b). When the data were adjusted for age, race, and ethnicity, higher DNA methylation was still associated with ER/PR-positive status across the genes studied. In age- and race/ethnicity-adjusted logistic regressions models of receptor-positive status against each gene mean methylation value individually, two thirds of the 806 associations were qualitatively positive (0.68; 95 % CI 0.66, 0.72) (Table 2). This represented twice as many positive associations compared with inverse associations (ratio of positive to inverse associations = 2.2; 96 % CI 1.9, 2.6). When restricting analyses to coefficients with *P* values not exceeding 0.05, more than 80 % of the 46 remaining associations were positive associations (0.83; 95 % CI 0.70, 0.93). This represented nearly five times as many positive associations compared with inverse associations (ratio of positive to inverse associations = 4.8; 96 % CI 2.4, 13).

**Table 2:** Number of associations between DNA methylation and hormone receptor-positive breast cancer in the BCCC dataset

Analysis	Number of coefficients^a^	No. of positive associations	No. of inverse associations	% positive^b^	95 % CI	Ratio^c^	95 % CI
Training dataset (*N* = 807 genes)							
All associations	806	548	258	68	(66, 72)	2.2	(1.9, 2.6)
Associations with *P* value ≤0.20	180	146	34	81	(75, 86)	4.3	(3.0, 6.4)
Associations with *P* value ≤0.05	46	38	8	83	(70, 93)	4.8	(2.4, 12.7)

^a^Number of logistic regression coefficients involved in each analysis

^b^Percentage of coefficients that represent a positive association between methylation and hormone receptor-positive breast cancer.

^c^Ratio of the number of positive divided by the number of negative associations

### Association of ER/PR status with DNA methylation at specific genes

In order to identify genes which promoter DNA methylation is associated with hormone receptor status, we conducted significance analysis of microarrays (SAM) analysis. The top 25 genes that had higher levels of DNA methylation associated with receptor-positive disease (Table 3, *left* column) were selected for more detailed analysis. Hypermethylation of *FZD9*, *MME*, *RAB32*, *BCAP31*, *HDAC9*, *PAX6*, *SCGB3A1*, *PDGFRA*, *IGFBP3*, *PTGS2*, and *SRC* were the strongest predictors of ER/PR-positive disease in the training dataset. Notably, none of the top performing 25 genes had positive associations between methylation and ER/PR-negative disease. To relate the predicted value of the identified genes, we performed hierarchical clustering of *β* values for all genes on the platform. In contrast to the predictor genes, hierarchical clustering of *β* values using the whole set of GoldenGate genes was unable to group samples according to the receptor status (Additional file 1: Figure S1).

**Table 3:** Validation of genes with differential DNA methylation as predictors of hormone status from the Illumina BCCC (training dataset) using TCGA (validation dataset)

	Training dataset		Validation dataset		Correlation with expression	
Gene	Association^a^	SAM *d* score^b^	Association^a^	SAM *d* score^c^	*ρ* ^d^	*P* value^d^
*FZD9*	Positive	3.94	Positive	8.65	−0.44	1.4E−15
*MME*	Positive	2.95	Positive	2.14	−0.13	2.1E−02
*RAB32*	Positive	2.70	Positive^e^	1.11	−0.27	1.4E−06
*BCAP31*	Positive	2.66	Positive	5.61	−0.28	4.9E−07
*HDAC9*	Positive	2.64	Positive	7.60	−0.15	8.5E−03
*PAX6*	Positive	2.64	Positive	4.56	−0.27	2.0E−06
*SCGB3A1*	Positive	2.53	Positive	9.51	−0.29	3.8E−07
*PDGFRA*	Positive	2.52	Positive	2.09	−0.30	1.1E−07
*IGFBP3*	Positive	2.51	Positive	6.37	−0.22	1.1E−04
*PTGS2*	Positive	2.50	Positive	5.69	−0.30	5.2E−08
*SRC*	Positive	2.50	Not-associated	0.00	NA	NA
*CHI3L2*	Positive	2.45	Positive	2.65	−0.69	2.2E−44
*PGR*	Positive	2.44	Positive	5.39	0.34	1.3E−09
*TMPRSS4*	Positive	2.43	NA	NA	NA	NA
*RASSF1*	Positive	2.43	Positive	7.78	−0.05	4.2E−01
*TBX1*	Positive	2.43	Positive	4.62	−0.05	4.2E−01
*PARP1*	Positive	2.38	Positive	2.48	−0.12	2.0E−02
*COL1A1*	Positive	2.32	Positive	4.15	0.08	1.7E−01
*SOX17*	Positive	2.32	Positive	2.22	−0.13	5.7E−05
*RUNX3*	Positive	2.29	Positive	7.06	−0.13	2.0E−02
*TES*	Positive	2.23	Positive	2.15	−0.45	2.6E−16
*GPATC3*	Positive	2.21	Positive^e^	0.17	NA	NA
*S100A2*	Positive	2.21	Positive	9.32	−0.52	2.6E−22
*MYH11*	Positive	2.20	Positive	3.61	−0.10	8.0E−02
*BMP2*	Positive	2.19	Positive	4.66	−0.37	1.3E−11

*NA* gene was absent in the dataset

^a^Indicates whether gene hypermethylation was associated with increased likelihood of ER/PR-positive breast cancer versus ER/PR-negative breast cancer (“Positive”)

^b^ *d* scores from SAM analysis using Δ of 0.7 on the GoldenGate dataset

^c^ *d* scores from SAM analysis using Δ of 3 on the TCGA dataset. In cases where several probes per gene were present, the data is shown for the probe with the highest SAM *d* score

^d^Pearson correlation coefficient between methylation and expression from TCGA and the corresponding *P* value

^e^Non-significant association

### DNA methylation at disease predictor genes in the validation dataset

We examined whether the 25 predictive gene methylation markers identified through the BCCC study (training dataset) would predict hormone receptor status in data from The Cancer Genome Atlas (TCGA) (validation dataset). Methylation data from TCGA represent a much larger platform, with 27,578 probes corresponding to 14,475 genes in total [12]. An ER/PR-specific DNA methylation pattern was apparent in these data from TCGA (Additional file 1: Figure S2). The prevalence of ER/PR-positive disease in the TCGA validation dataset (78 %, 239 of 306) (Table 4) was similar to that in our training dataset (71 %, 53 of 75) (Table 1). We performed analysis of the TCGA data similar to the BCCC data (Additional file 1: Figure S3). In the validation (TCGA) dataset, 2088 DNA methylation probes were strongly associated (*d* value >3.5) with ER/PR status. Multiple probes for *IGFBP3* or *PTGS2* showed similar DNA methylation pattern (Additional file 1: Figure S4). Other predictor genes such as *RASSF1*, however, had a subset of probes that did not distinguish between ER/PR-positive and ER/PR-negative disease. The number of genes exhibiting hypermethylation in ER/PR-positive tumors was five times larger than the number of genes exhibiting hypermethylation in ER/PR-negative tumors.

**Table 4:** Patient and tumor characteristics by hormone receptor status in the TCGA dataset

	Total (*N* = 306)		ER/PR positive	
	*N*	%	%	*P* value (chi-square test)
Age at diagnosis				0.008
<50	72	24	68	
50–59	79	26	73	
60–79	155	51	85	
Race/ethnicity				0.001
nH White	182	59	77	
nH Black	22	7	59	
Hispanic	1	0	100	
Asian	21	7	57	
Unknown	80	26	91	
Pathological stage				0.271
1	55	18	82	
2	180	59	84	
3	51	17	74	
4	9	3	80	
Missing	11	4	100	

**Fig. 2:**
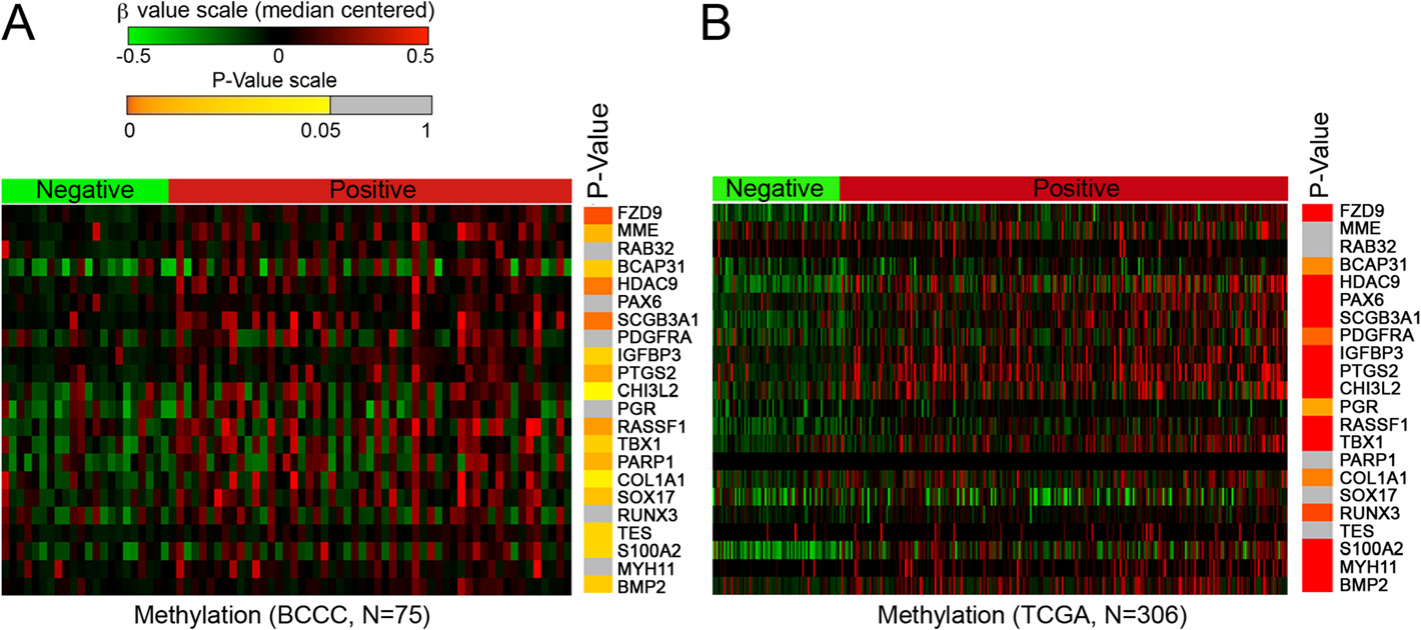
Training (BCCC) and validation (TCGA) datasets stratified based on DNA methylation data for disease predictor genes. **a** Sample level data for DNA methylation (GoldenGate) in the BCCC dataset. Results of SAM-supervised classification of ER/PR status from *β* values are shown. *β* values for disease predictors (one probe per gene listed in Table 3) are presented as a heat map. Data are shown grouped for hormone receptor-negative samples (*N* = 22) and hormone receptor-positive samples (*N* = 53). *P* values from the *t* test for the difference between ER/PR-negative and ER/PR-positive disease (Additional file 1: Table S1) are presented on the *right*. **b** Sample level data for DNA methylation in the TCGA. Mean *β* values (when multiple probes were present) are reported for Table 3 genes, stratifying samples according to ER/PR status. *P* values from the *t* test are shown on the *right*

The BCCC dataset and the TCGA dataset were generated using different high-throughput platforms, with distinct probe design. Matching of the features from different platforms can be approached in different ways. When we matched the DNA methylation data at the gene level, associations between methylation and ER/PR status observed in the training dataset were generally reproduced in the validation dataset (Fig. 2). Despite the differences between the two platforms and patient cohorts, strong correlations between DNA methylation level and ER/PR status were observed for 21 out of the 24 gene methylation markers identified in the training dataset with methylation data in the validation dataset (Table 3); 17 were identified as predictors in the validation dataset based on *t* tests (Fig. 2 and Additional file 1: Table S1). These results revealed a high degree of consistency between the BCCC dataset and the validation TCGA dataset.

### DNA methylation at predictor genes correlates with gene expression level

**Fig. 3:**
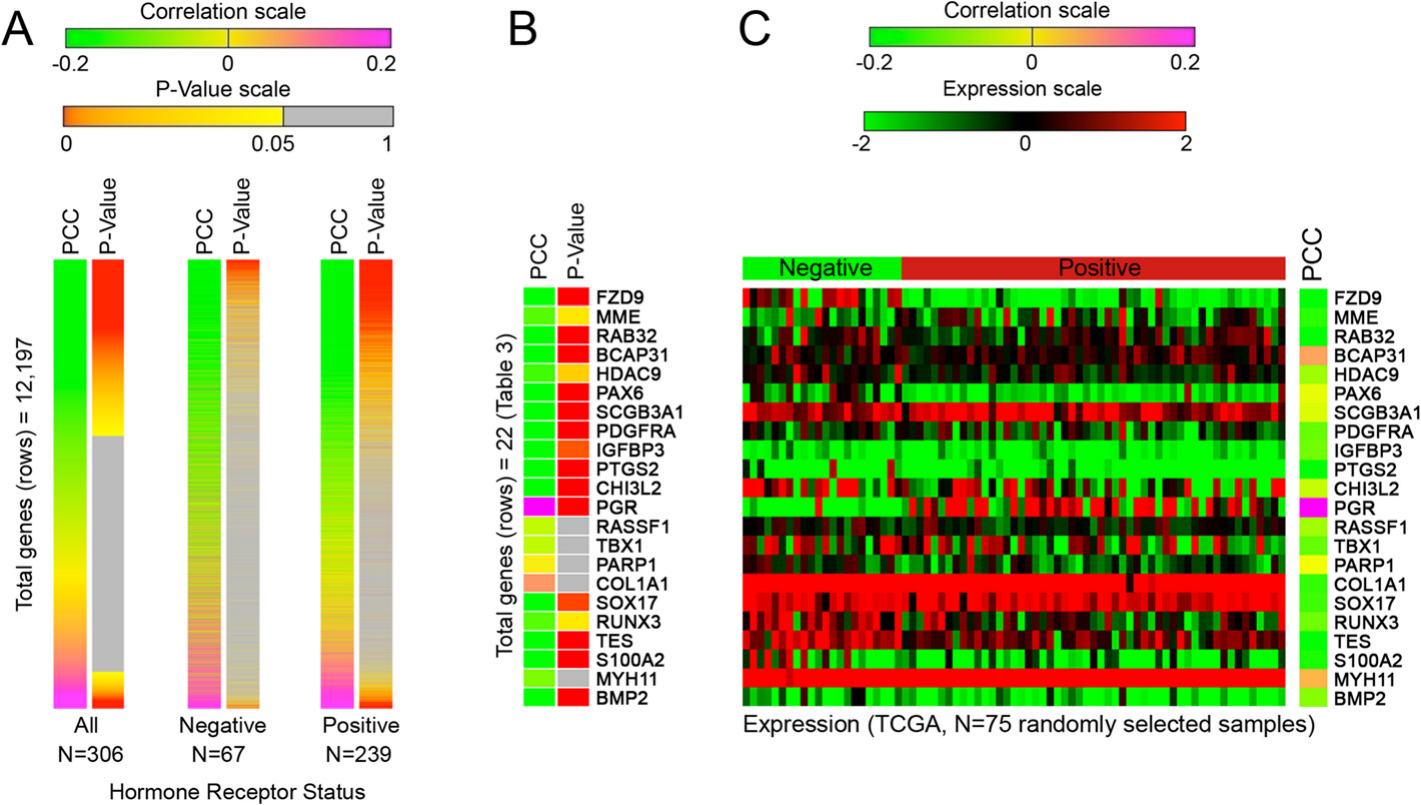
Correlation of DNA methylation with the level of gene expression in the validation dataset (TCGA). **a** Significance of the correlation between the level of DNA methylation and expression for each gene as determined by Pearson correlation coefficient (PCC). The correlation test was run for all samples, only ER/PR-negative samples, or only ER/PR-positive samples. **b** Correlation test (methylation versus expression) for predictors (Table 3). **c** Sample level data for gene expression of predictors (Table 3). Data is presented for 22 ER/PR-negative tumors and 53 ER/PR-positive tumors. The PCC across the BCCC samples and TCGA samples (methylation versus expression) is shown on the *right*

Within the TCGA (validation) dataset, both DNA methylation and gene expression data were available for a total of 12,197 genes. Consistent with the generally inhibitory effect of DNA methylation on gene activity, DNA methylation was generally inversely correlated with messenger RNA (mRNA) expression regardless of ER/PR status (Additional file 2: Table S2, *P* value <0.05 in Additional file 1: Figure S5). The number of genes for which expression was significantly inversely correlated with DNA methylation was twofold higher among ER/PR-positive than among ER/PR-negative tumors (5649 versus 2771, respectively, *P* value <0.05 in Fig. 3a), suggesting that ER/PR-positive tumors have more stable level of expression at genes experiencing DNA methylation than ER/PR-negative tumors.

Many of the gene methylation markers that predicted of ER/PR status in the training dataset of 75 samples showed an inverse correlation between DNA methylation and gene expression level when analyzed in sets of 75 randomly assembled TCGA samples (Additional file 1: Figure S6). Regardless of whether we used DNA methylation data from the training or the validation dataset, an inverse correlation was common for gene methylation markers that were predictors of ER/PR status (compare Fig. 3b, c, also see Additional file 1: Figure S7). Highly significant inverse correlations were observed for *FZD9*, *HDAC9*, *PAX6*, *PDGFRA*, *S100A2*, and *BMP2* genes, suggesting that DNA methylation at these CpG sites results in stable changes in gene expression. A positive correlation between DNA methylation and expression was found for one gene (i.e., *PGR*), suggesting that DNA methylation at the CpG sites within the *PGR* gene that were analyzed are not relevant to regulation of promoter activity. Sample level data showed that the predictor genes differ in the absolute level and robustness of changes in gene expression between the ER/PR-positive and ER/PR-negative groups (Fig. 3c). Our results suggest that hormone status correlates with DNA methylation status and with the activity of the identified set of genes.

## Discussion

In a training dataset of newly diagnosed breast cancer patients, we observed a general tendency for higher levels of DNA methylation to be associated with ER/PR-positive disease, and we identified a set of predictor genes for which hypermethylation was highly significantly associated with ER/PR-positive disease. The vast majority of the predictor genes were confirmed in a validation dataset despite the fact that the methylation data from the validation dataset relied on a mostly different set of probes within the same 24 genes. Finally, increased methylation was associated with reduced expression for the vast majority of gene methylation markers, suggesting that we had identified a reproducible set of genes whose methylation might play an etiologic role in breast cancer subtypes.

Differential DNA methylation according to ER/PR status has previously been observed in breast cancer in both genome-wide studies [8–10, 12] and in studies of individual genes [13, 14]. In contrast, there was only a modest association of DNA methylation with HER2-positive status [12]. A large study of 466 breast cancers found that basal-like cancers which were 90 % ER/PR negative had a tendency to display hypomethylation, while luminal B (ER/PR-positive) breast cancers had a tendency to display hypermethylation [12]. The luminal B group had a low rate of mutations, in contrast to the group with hypomethylation that had p53 mutations. The characteristic hypermethylation of CpG islands related to ER/PR-positive breast tumors, which was termed “breast CpG island methylator phenotype” (B-CIMP), was first reported in 2011 [15]. The phenomenon of methylator phenotype was associated with low risk of metastasis and high rates of survival independently of known breast cancer characteristics. This result is consistent with the finding presented here that DNA methylation was generally associated with less aggressive, ER/PR-positive breast cancer.

The predictor genes that were differentially methylated according to ER/PR status were also differentially expressed. Loss of DNA methylation is not a prerequisite of increase in gene activity [16], and genomic regions that are hypomethylated in breast cancer cells compared to normal mammary epithelial cells do not necessary exhibit an increase in expression [17]. DNA hypomethylation occurs primarily in the form of partially methylated domains, displaying allelic DNA methylation, where one allele is DNA methylated while the second allele exhibits histone methylations H3K9me3 or H3K27me3 [17]. Because these histone modifications support the formation of repressive chromatin, loss of DNA methylation at one allele while retaining H3K9me3 or H3K27me3 at another allele fails to activate gene expression. On another hand, acquisition of DNA methylation at a single allele may have an effect on gene activity by limiting chromatin accessibility and transcription factor binding even in the absence of repressive histone modifications at another allele. The analysis that we report in this paper does not address the exact process through which DNA methylation might contribute to the repression of gene expression.

Our study yielded 24 disease predictors, which all have been linked to cancers in previous studies. When arranged in the order of significance (i.e., *d* value) from the top to the bottom genes in the list, the top gene is a receptor for Wnt signaling, frizzled class receptor 9 (*FZD9*). Hypermethylation of *FZD9* has been previously shown to be associated with ER+ tumors [8, 10]. Hypermethylation of *FZD9* correlates with transcriptional repression and is an independent predictor of poor prognosis for patients with acute myeloid leukemia (AML) [18, 19]. The bottom gene in the list encodes Bone Morphogenetic Protein 2 (BMP2). Promoter DNA methylation of *BMP2* contributes to drug resistance in breast cancer [20]. Another most commonly inactivated gene in various cancers, Ras association domain-containing protein 1, *RASSF1*, undergoes either DNA methylation or chromosomal deletion in breast cancer [21]. In patients with chromosome deletion of *FZD9* or *RASSF1*, aberrant methylation of the remaining allele was associated with the poorest clinical outcome [19, 21], indicating their functional contribution.

If developed as diagnostic tool in breast cancer, DNA methylation has certain advantages over other epigenetic biomarkers. DNA methylation pattern is preserved under harsh conditions and requires small amount of the sample, which is important in clinical practice, whereas microRNAs and histone modifications require robust high-quality material. In addition, DNA methylation markers may be detectable in plasma providing for development of non-invasive techniques for early detection and follow-up of breast cancer. For example, aberrant DNA methylation of *SOX17* has been identified not only in tumor tissue but also in plasma DNA [22], and *SOX17* appears to be hypermethylated in luminal B tumors, but hypomethylated in basal-like tumors [23]. Genes such as *SOX17* thus could be used as a prognostic biomarker to identify patients at risk of developing metastasis or recurrence.

There are at least two other alternative explanations for the observed associations of gene DNA methylation with hormone receptor status in our study. First, specific methylation patterns may arise in tumors with different cell-type of origin, in which case, methylation might not be an etiologic driver of subtype but rather a marker of subtype. Second, other molecular events such as histone modifications associated with ER/PR may play an active role in dictating DNA methylation level, either globally or at specific genes. Traditionally, prediction of breast cancer survival has made use of ER/PR status. Recent effort towards integrated view of epigenomic features and transcriptome has provided important insights into population-based molecular subgrouping in several cancer types [24]. Defining such subgrouping in breast cancer and focusing further analysis on representative numbers from groups stratified by predictor genes will help to link ethnic and socioeconomic factors to etiology of ER/PR-positive and ER/PR-negative disease.

## Conclusions

We identified a set of genes in a genome-wide study whose DNA methylation status predicted ER/PR status in training dataset as well as in a validation dataset from TCGA. The patient cohorts were different in racioethnic distribution but nevertheless displayed the same predictor genes. Moreover, aberrant methylation for many of the genes identified in the present study has been found in breast or other cancers in prior studies, indicating their potential use as biomarkers. Increased methylation was associated with reduced expression for the vast majority of these genes, suggesting that it might play an etiologic role in breast cancer subtypes and may provide insights into biological pathways associated with tumors of particular hormone receptor status.

## Methods

### Training dataset

Formalin-fixed, paraffin-embedded (FFPE) tumor samples came from the Breast Cancer Care in Chicago (BCCC) study which has been described elsewhere [25]. The protocol for conducting this study has been approved by the University of Illinois at Chicago Institutional Review Board, and details on the consent process have been published [26]. Association of clinicopathological features with ER/PR status in the BCCC dataset was determined by chi-square test, and the *P* values are presented in Table 1. Copies of pathology reports and the corresponding set of hematoxylin and eosin (H&E)-stained slides were requested from the pathology department at each diagnosing institution, and a single pathologist selected tumor blocks representative of the tumor. Two recuts (at 4 μm each) were made from each selected block for H&E staining. The recuts were then examined in order to identify invasive components of the sample, and areas were marked according to tissue component. Cores of invasive tissue (2 mm in diameter) were obtained from the marked areas.

DNA extraction was performed by adding to each core 100 μl xylene. After the incubation with gentle shaking for 5 min, supernatant was removed by centrifugation at 14,000*g* to remove the paraffin. The process was repeated two more times. The tissue was then weighted and 2–4-mg tissue was used for DNA extraction using Gentra Puregene kit (QIAGEN). All tissues were homogenized after adding cell lysis solution, proteinase K, and overnight incubation. The extracted DNA was measured by NanoDrop and normalized at 50 ng/μl concentration. Bisulfite conversion was performed on 500 ng of extracted DNA using EZ DNA methylation kit (Cat # D5001, Zymo Research, Irvine, CA) according to the manufacturer’s instructions. As the result of conversion, unmethylated cytosine residues were converted to uracils. The converted DNA was eluted in 10 μl M-Elution buffer provided in the kit. DNA methylation assays were performed using 5 μl bisulfite-converted DNA in Illumina’s GoldenGate Assay for Methylation as per Illumina’s protocol. The converted DNA was biotinylated, and the allele-specific oligos were added (for methylated and unmethylated sequence). Unhybridized oligos were washed away, and hybridized oligos were enriched by PCR and hybridized to Sentrix Array Matrix Universal Probe Set 7A, 1536 Bead Types. Imaging was performed in the Bead Array Reader. The raw data was processed by the BeadStudio Methylation Module to generate *β* values. Eighty tumor samples were assayed, from which 75 were from patients that had information on ER/PR status, and these 75 samples were subjected to downstream analysis.

### Methylation assay using the GoldenGate platform

The GoldenGate Assay for Methylation (Illumina, San Diego, CA) is a high-throughput bisulfite- and ligation-based assay to detect DNA methylation from bisulfite-converted genomic DNA. The GoldenGate Methylation Cancer Panel I spans 1505 CpG loci selected from 807 genes. Each gene is represented by either one (28.6 %), two (57.3 %), or three and more (14.1 %) CpG sites. Approximately two thirds of analyzed CpG sites are contained within CpG islands (10). Each gene was represented by up to five CpG sites that were located in the promoter region. Genes included tumor suppressor genes, oncogenes, genes involved in DNA repair, cell cycle control, differentiation, and apoptosis. Information on genes contained in the GoldenGate Cancer Panel 1 can be found in a Gene Annotation Data File (available at http://support.illumina.com/downloads/goldengate_methylation_cancer_panel_product_files.html), including gene identification numbers, symbols, and synonyms.

### Mean gene methylation and receptor status

We modeled using logistic regression analysis each of the 807 mean gene methylation values as a predictor of ER/PR status (either positive versus both negative) one at a time while adjusting for age and race/ethnicity (nH White as the referent, nH Black, and Hispanic). The resulting logistic coefficients for methylation variables and their corresponding *P* values were assembled. We then dichotomized coefficients into those representing a qualitatively inverse association and those representing a qualitatively positive association of higher methylation with ER/PR-positive breast cancer. The percentage of associations that were positive, along with the ratio of the number of positive divided by the number of negative associations, was estimated along with 95 % confidence intervals using bootstrapped bias-correction procedures based on 1000 replications. In addition, to assess whether or not difference between DNA methylation was significantly different between ER/PR-positive and ER/PR-negative groups, two-sided *t* tests were conducted using the *β* value of each gene across the samples in these two groups.

### Validation dataset (TCGA) and HM27 platform

Association of clinicopathological features with ER/PR status in the TCGA dataset was determined by chi-square test, and the *P* values are presented in Table 4. DNA methylation and expression data archived within The Cancer Genome Atlas (TCGA) have been previously described [12] and were downloaded from the TCGA Data Portal website (http://tcga-data.nci.nih.gov/tcga/). We identified 306 receptor-positive and receptor-negative breast tumors from TCGA with data based on the Illumina Infinium DNA methylation platform, HumanMethylation27 (HM27) BeadChip (Illumina). The HM27 BeadChip contains 27,578 CpG sites in the proximity of transcription start sites for 14,475 genes in the NCBI Genome Build 36. The genomic locations and sequences for probes on the array were downloaded from the TCGA Data Portal. There were 720 genes in the HM27 data that overlapped with the probes from the GoldenGate. The TCGA dataset contains only a few specific probes from the GoldenGate; therefore, direct comparison at the probe level was not feasible between these two platforms. Thus, the validation dataset of *β* values was collected for all CpG sites corresponding to the genes defined as predictors in training dataset (Table 3). From 25 predictor genes identified with GoldenGate, TCGA contained data for 24 of these. Tumors from the TCGA dataset were also analyzed for gene expression; these data were generated on Agilent custom 244K whole genome microarrays.

### Using significance analysis of microarrays for association of differential DNA methylation to hormone receptor status

For both the training (BCCC) and validation (TCGA) datasets, the level of methylation at each CpG site was defined by a *β* value, with 0 indicating 0 % DNA methylation and 1 indicating 100 % DNA methylation. All *β* values for CpG sites that annotated to the same gene were averaged, and each mean gene methylation variable was modeled as a predictor of ER/PR status. Analysis was performed using significance analysis of microarrays (SAM) algorithm (http://statweb.stanford.edu/~tibs/SAM/). SAM uses permutations of the data in order to identify a threshold that can be used to control the false discovery rate (FDR). The following parameters were used: S0 parameters were selected using Tusher et al. method and K-nearest neighbor imputer was used with 10 neighbors.

### mRNA gene expression profiling dataset (TCGA)

Tumors from the TCGA dataset were analyzed for gene expression using data generated on Agilent custom 244K whole genome microarrays. For correlation analyses, genes for which there were no expression values recorded were removed from the analysis. Thus, 12,197 genes were available to analyze the correlation between methylation and expression, including 720 of the 807 genes of the GoldenGate platform. Kolmogorov-Smirnov (ks) tests, Wilcox tests, and Pearson correlation coefficients (PCC) were calculated using R/Bioconductor software (http://www.bioconductor.org). The heat maps were generated using GiTools [27].

### Availability of supporting data

The datasets supporting the results of this article are available at the Gene Expression Omnibus series GSE72110.

The datasets supporting the results of this article are also included as its additional files. Additional file 1: Table S1 and Figures S1–S7. Additional file 2: Table S2 listing the PCC with associated *P* value for genes in the TCGA dataset to assess correlation between DNA methylation and mRNA expression.

## Additional files

Additional file 1:
**Table S1 and Figure S1–S7. Table 1.** Results of the two-sided *t* test analysis for receptor positive and negative disease for gene methylation markers in the training dataset and TCGA (validation dataset). **Figure S1.** Hierarchical clustering (HCL) of DNA methylation data to identify groups of patients with ER/PR-positive disease. **Figure S2.** Results of SAM supervised classification of ER/PR status from *β* values using TCGA dataset. **Figure S3.** Integration of three platforms. **Figure S4.** Heat map of *β* values for individual probes of genes in **Table** **3.** **Figure S5.** Correlation of DNA methylation with the level of gene expression across the genes in each tumor in TCGA data. **Figure S6.** Correlation of GoldenGate DNA methylation data with the level of expression for each gene across randomly selected tumors. **Figure S7.** Correlation of GoldenGate DNA methylation data with the level of expression for disease predictor genes. (PDF 8112 kb) Additional file 2: Table S2. Correlation between the level of methylation and gene expression as determined by Pearson correlation coefficient (PCC). (XLSX 518 kb)

## Abbreviations

BCCC: Breast Cancer Care in Chicago
ER/PR: estrogen (ER) and progesterone (PR) receptors
TCGA: The Cancer Genome Atlas
