# Supplementary material for: DNA methylation and hormone receptor status in breast cancer
Source: Clin Epigenetics. 2016 Feb 16;8:17. doi: 10.1186/s13148-016-0184-7 (PMC4754852; doi:10.1186/s13148-016-0184-7)
Supplement: Additional file 1: — Table S1 and Figure S1–S7. Table 1. Results of the two-sided t test analysis for receptor positive and negative disease for gene methylation markers in the training dataset and TCGA (validation dataset). Figure S1. Hierarchical clustering (HCL) of DNA methylation data to identify groups of patients with ER/PR-positive disease. Figure S2. Results of SAM supervised classification of ER/PR status from β values using TCGA dataset. Figure S3. Integration of three platforms. Figure S4. Heat map of β values for individual probes of genes in Table 3. Figure S5. Correlation of DNA methylation with the level of gene expression across the genes in each tumor in TCGA data. Figure S6. Correlation of GoldenGate DNA methylation data with the level of expression for each gene across randomly selected tumors. Figure S7. Correlation of GoldenGate DNA methylation data with the level of expression for disease predictor genes. (PDF 8112 kb) [file 13148_2016_184_MOESM1_ESM.pdf]

SUPPLEMENTAL TABLE 1. Results of the two-sided T-test analysis for receptor positive and negative disease for gene methylation markers in the training dataset and TCGA (validation dataset).

	Training Dataset	Validation Dataset
Gene	T-TEST P-Value	T-TEST P-Value
FZD9	8.62E-05	5.00E-16
MME	3.85E-03	1.22E-01
RAB32	6.49E-02	1.70E-01
BCAP31	7.68E-03	6.96E-04
HDAC9	3.86E-04	0.00E+00
PAX6	6.88E-02	1.73E-09
SCGB3A1	3.19E-04	3.42E-14
PDGFRA	9.65E-02	1.87E-04
IGFBP3	9.41E-03	1.36E-10
PTGS2	1.99E-03	1.40E-10
CHI3L2	4.11E-02	3.92E-07
PGR	1.77E-01	2.10E-03
RASSF1	1.48E-03	1.34E-14
TBX1	8.44E-03	8.31E-08
PARP1	3.01E-03	7.25E-01
COL1A1	2.81E-02	4.96E-04
SOX17	5.27E-03	1.30E-01
RUNX3	1.91E-01	5.99E-05
TES	1.08E-02	1.99E-01
S100A2	1.15E-02	0.00E+00
MYH11	6.48E-02	7.45E-07
BMP2	7.94E-03	7.28E-08

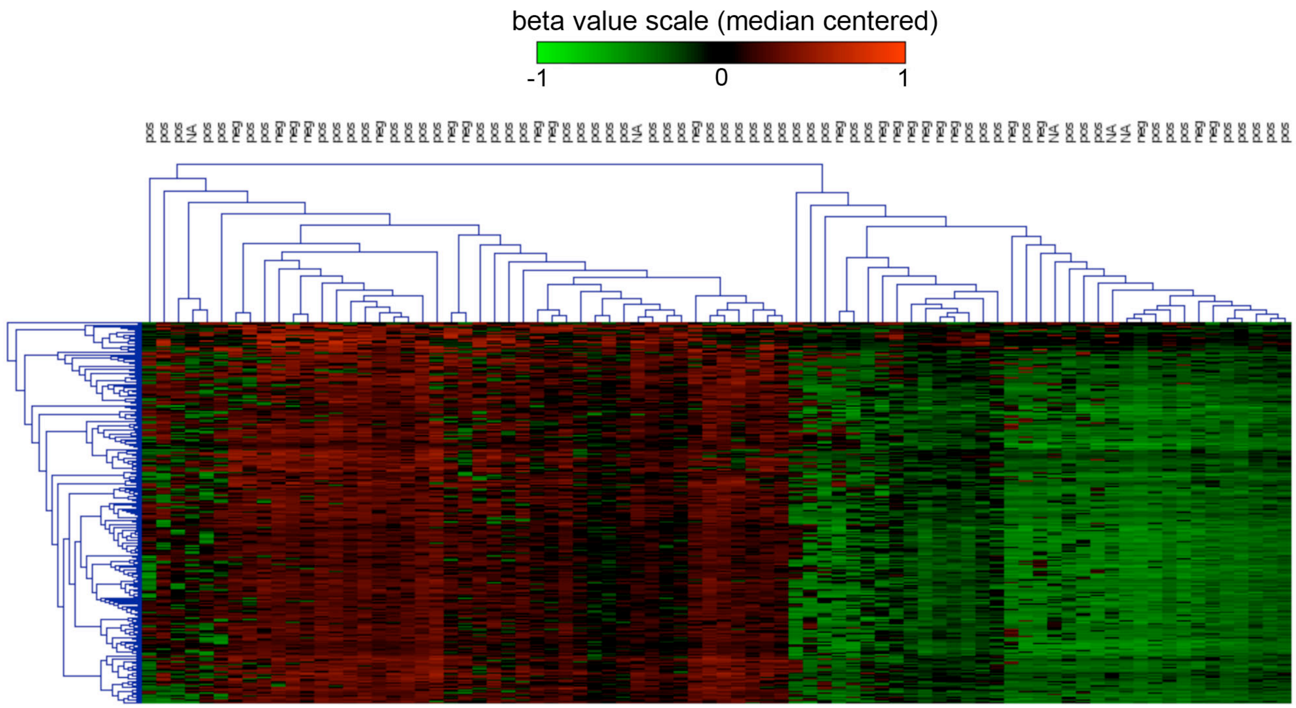

SUPPLEMENTAL FIGURE S1. Hierarchical clustering (HCL) of DNA methylation data to identify groups of patients with ER/PR-positive disease. Using standard deviation (SD) filter, we included probes that vary in β values across the samples. In particular, we have taken only those probes that vary >= 0.2 SD. The total of 270 genes passed this criteria. Median centered β values for these genes were subjected to Euclidean clustering. “neg” – ER/PR negative sample, “pos” – ER/PR positive sample, and “NA” –no information available for the sample.

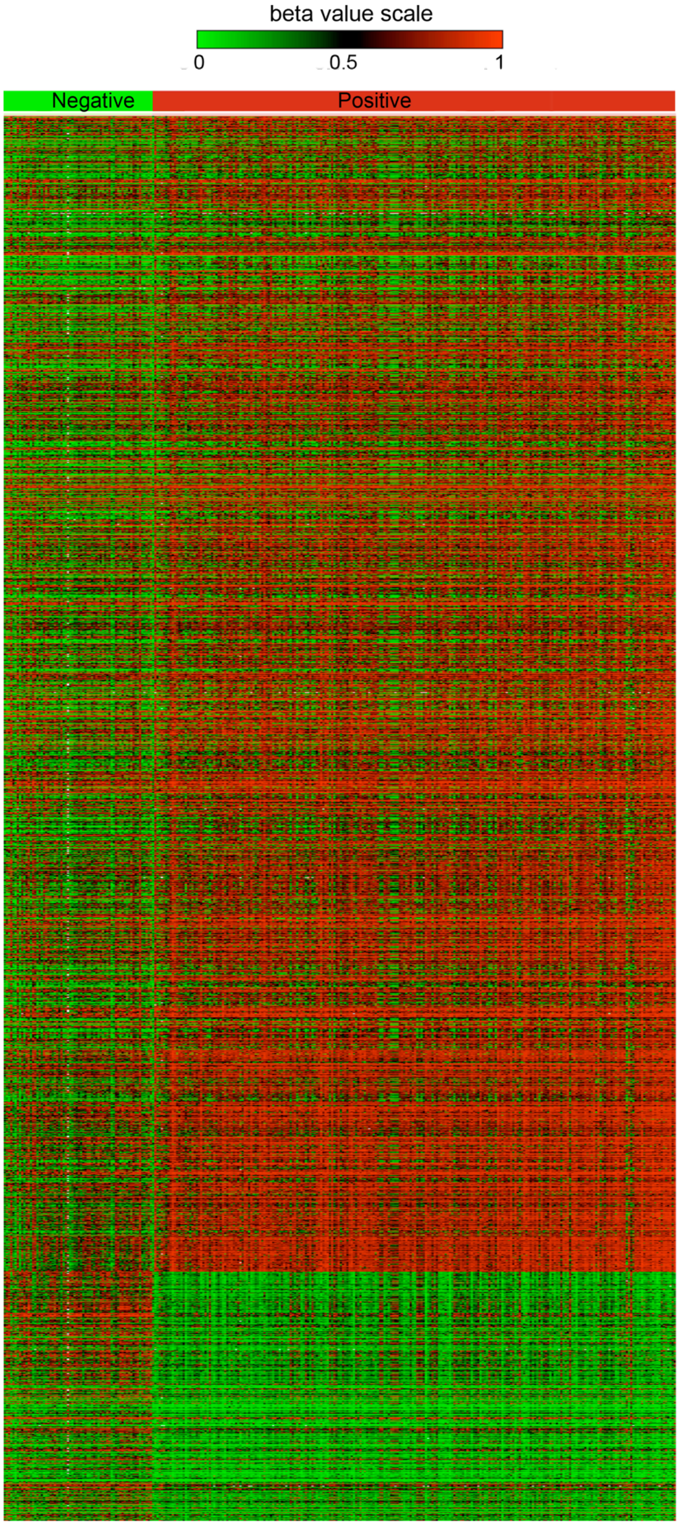

Methylation (TCGA, N=306 samples)

SUPPLEMENTAL FIGURE S2. Results of SAM supervised classification of ER/PR status from β values using TCGA data set. SAM statistics with d value > 3.5 identified 2,088 probes, corresponding to 1,748 unique differentially methylated genes. Data are shown grouped for ER/PR negative

samples (N=67) and ER/PR positive samples (N=239).

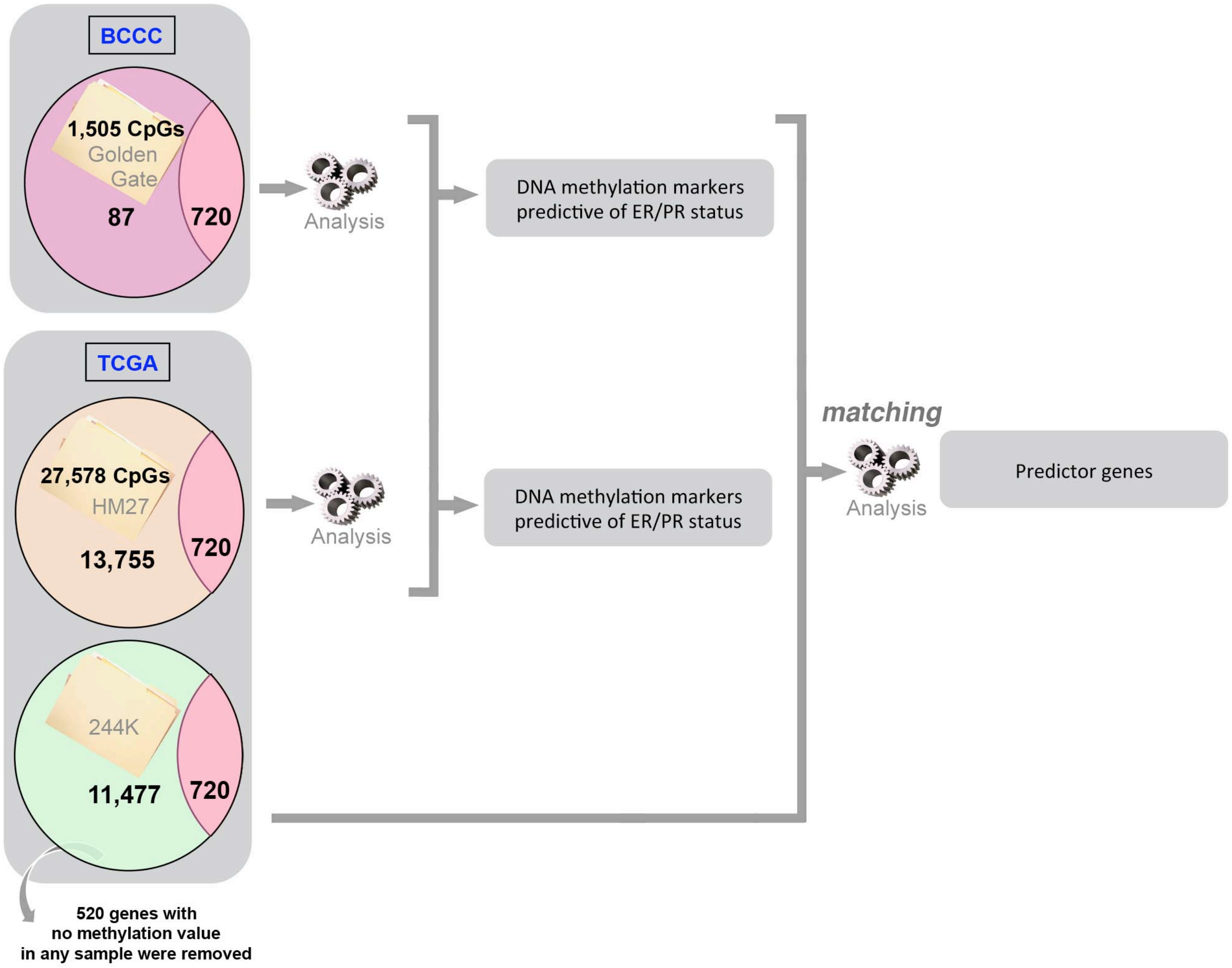

SUPPLEMENTAL FIGURE S3. Integration of three platforms. Two platforms, DNA methylation and gene expression, were used from the TCGA. The total number of intersecting genes between the BCCC platform and TCGA platforms is 720 and shown as a Venn diagram next to the number of other features under the platform name. The DNA methylation markers predictive of ER/PR status were determined for two platforms and matched individually between all three platforms.

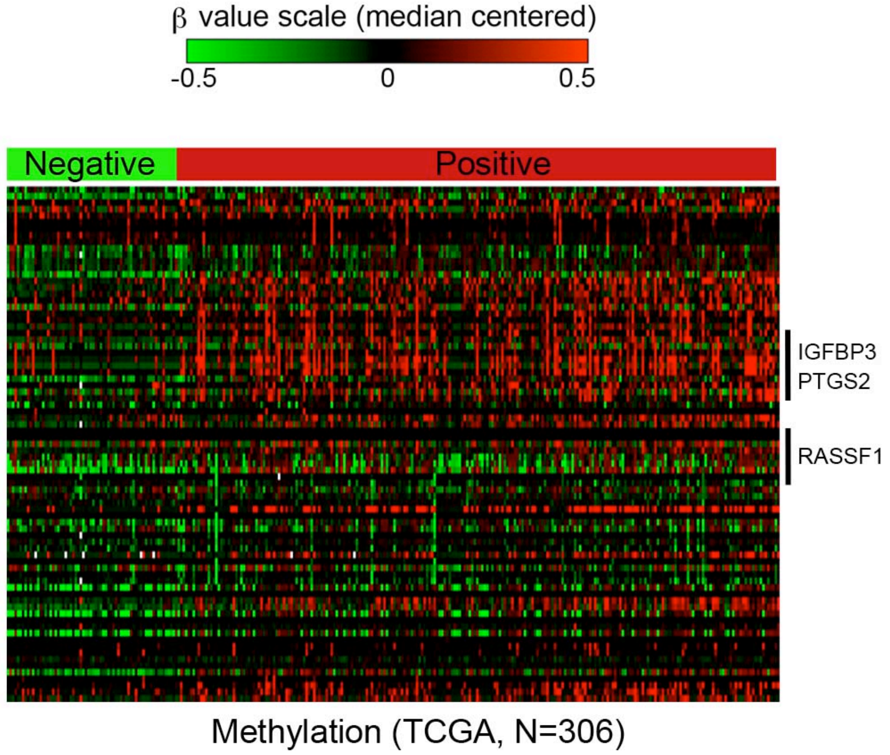

SUPPLEMENTAL FIGURE S4. Heat map of β values for individual probes of genes in Table 3. Data are grouped for ER/PR negative samples (N=22) and ER/PR positive samples (N=53).

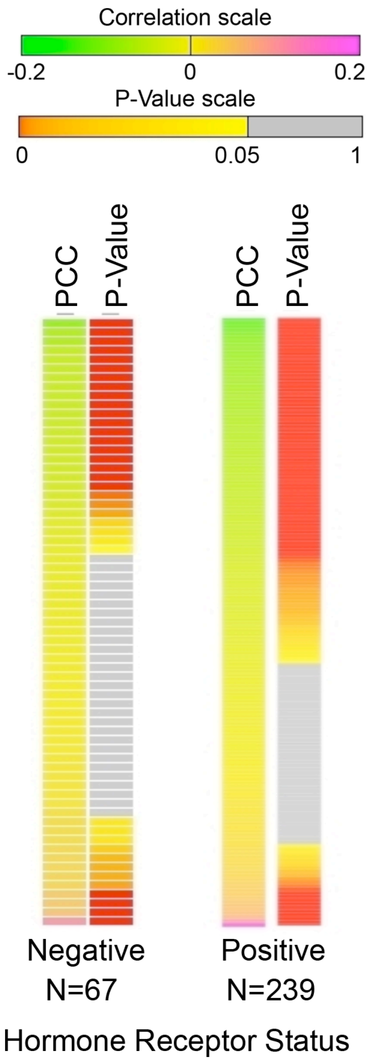

SUPPLEMENTAL FIGURE S5. Correlation of DNA methylation with the level of gene expression across the genes in each tumor in TCGA data. Significance of the correlation between DNA methylation and expression data values for all 12,197 genes in each tumor sample is presented by Pearson Correlation Coefficient (PCC). The correlation test (methylation versus expression) was run for 67 ER/PR negative and 239 ER/PR positive samples.

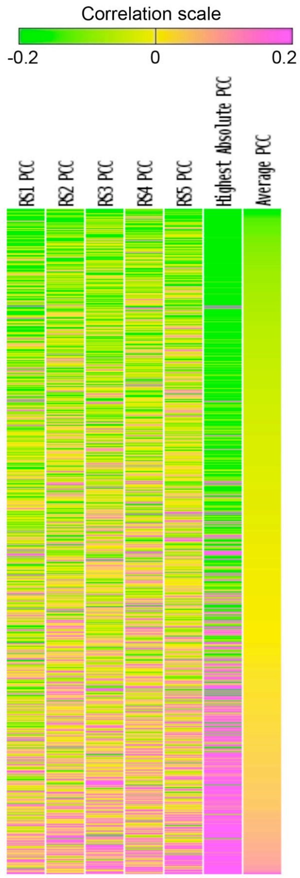

SUPPLEMENTAL FIGURE S6. Correlation of GoldenGate DNA methylation data with the level of expression for each gene across randomly selected tumors. PCC was performed for 720 genes present at the GoldenGate platform. Because TCGA tumor set is larger than ours, 75 tumor samples from our data set were compared to five randomly selected (RS1-RS5) TCGA groups of 75 tumor samples. All groups of 75 samples contained 22 ER/PR negative and 53 ER/PR positive samples like our dataset. Average PCC represents average of PCCs for five individual TCGA groups of 75 samples. Highest PCC represents the highest PCC value observed among the five groups.

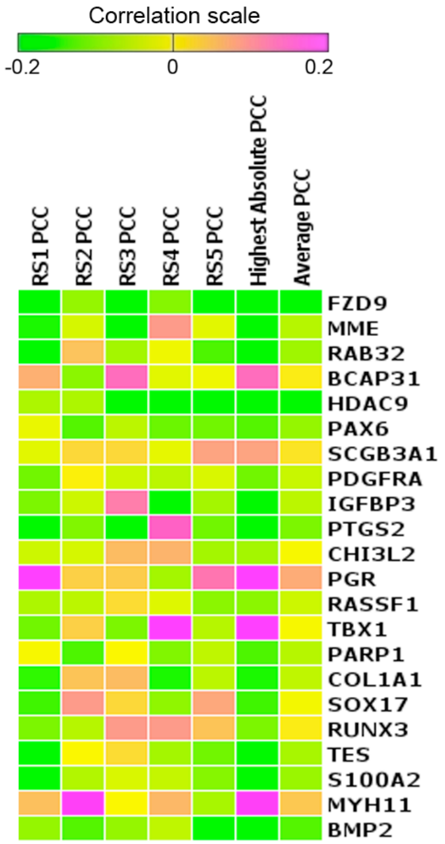

SUPPLEMENTAL FIGURE S7. Correlation of GoldenGate DNA methylation data with the level of expression for disease predictor genes. Correlation test (methylation versus expression) for each gene from Table 3 is shown across the TCGA samples. Methylation values for our set of 75 samples were correlated with expression values in five randomly selected TCGA sets of 75 samples.
